# The N-terminal domain of *Escherichia coli *RecA have multiple functions in promoting homologous recombination

**DOI:** 10.1186/1423-0127-16-37

**Published:** 2009-04-01

**Authors:** Chien-Der Lee, Ting-Fang Wang

**Affiliations:** 1 Institute of Molecular Biology, Academia Sinica, Taipei 115, Taiwan

## Abstract

*Escherichia coli *RecA mediates homologous recombination, a process essential to maintaining genome integrity. In the presence of ATP, RecA proteins bind a single-stranded DNA (ssDNA) to form a RecA-ssDNA presynaptic nucleoprotein filament that captures donor double-stranded DNA (dsDNA), searches for homology, and then catalyzes the strand exchange between ssDNA and dsDNA to produce a new heteroduplex DNA. Based upon a recently reported crystal structure of the RecA-ssDNA nucleoprotein filament, we carried out structural and functional studies of the N-terminal domain (NTD) of the RecA protein. The RecA NTD was thought to be required for monomer-monomer interaction. Here we report that it has two other distinct roles in promoting homologous recombination. It first facilitates the formation of a RecA-ssDNA presynaptic nucleoprotein filament by converting ATP to an ADP-Pi intermediate. Then, once the RecA-ssDNA presynaptic nucleoprotein filament is stably assembled in the presence of ATPγS, the NTD is required to capture donor dsDNA. Our results also suggest that the second function of NTD may be similar to that of Arg243 and Lys245, which were implicated earlier as binding sites of donor dsDNA. A two-step model is proposed to explain how a RecA-ssDNA presynaptic nucleoprotein filament interacts with donor dsDNA.

## Background

*Escherichia coli *RecA is the founding member of the RecA protein family. It is essential for the initiation of repair of DNA breaks via homologous recombination, induction of the DNA damage-induced 'SOS' response, and activation of translesion DNA synthesis, as well as development and transmission of antibiotic resistance genes [1,2]. Nearly all known functions of RecA require the formation of a presynaptic helical filament comprised of single-stranded DNA (ssDNA) bound to multiple RecA monomers with ATP. During homologous recombination, this activated form of the helical filament is capable of interacting with homologous double-stranded DNA (dsDNA) to form a heteroduplex DNA molecule. Eventually, the DNA strands are exchanged, resulting in the displacement of one of the original duplex strands and the subsequent creation of a new heteroduplex (or D-loop). This function is evolutionarily conserved in other members of the RecA family, including archaeal RadA and the eukaryotic proteins, Rad51 and Dmc1.

The RecA monomer has three major structural domains: a small N-terminal domain (NTD), a core ATPase domain (CAD), and a large C-terminal domain (CTD). By contrast, the monomers of RadA/Rad51/Dmc1 consist of a CAD and a larger NTD. The CAD, often referred to as the RecA fold [3], is structurally similar to the ATPase domains of DNA/RNA helicases, F1 ATPases, chaperone-like ATPases, and membrane transporters [4]. Highly conserved in all RecA family proteins, the CAD contains two disordered loops (denoted the L1 and L2 motifs) that bind to ssDNA and are responsible for ssDNA-stimulated ATPase activity [5]. All RecA family proteins are polymerized via a polymerization motif located between the NTD and the CAD. The polymerization motif contains a hydrophobic residue (isoleucine 26 in *E. coli *RecA; phenylalanines in RadA, Rad51, and Dmc1) that docks within the hydrophobic pocket of the neighboring CAD. This interaction was also observed at the binding interface between a human Rad51 monomer and a BRC repeat of BRCA2 tumor suppressor protein [6-9]. The polymerization motif of archaeal RadA protein is responsible for the assembly of different quaternary structures, including toroidal rings, as well as right-handed and left-handed helical filaments [10,11].

The crystal structures of RecA-ssDNA and RecA-dsDNA nucleoprotein complexes with ADP-AlF_4_^-^-Mg^2+ ^have recently been reported [12]. These new structures have provided unprecedented new insights into the mechanisms and energetics of RecA protein. In the RecA-ssDNA-ADP-AlF_4_^-^-Mg^2+ ^filament complex, the ssDNA is bound by the L1 and L2 loop regions as well as by the N-terminal portion of the αF and αG helices that follow L1 and L2, respectively. The ADP-AlF_4_^-^-Mg^2+ ^is sandwiched between the CADs of two adjacent RecA protomers in a completely buried environment. AlF_4_^- ^group is coordinated by the side chains of Lys248 and Lys250. Lys250 also hydrogen bonds to the side chain of Glu96 in the neighboring RecA protomer. Glu96 is the catalytic residue thought to activate a water molecule for nucleophilic attack on the γ-phosphate. This second interface is absent in the inactive filament, where the corresponding interface of the ATP analogue, adenylyl-imidodiphosphate (AMP-PNP), is solvent exposed. Therefore, the charged-stabilized hydrogen bonds that Lys248 and Lys250 make to the AlF_4_^- ^group could explain the ATP-dependency of DNA binding, because the γ-phosphate of ATP is sensed across the RecA-RecA interface cooperating with DNA binding to promote the transition to the active filament state. Close to the filament axis, the ssDNA is extended 50% in length relative to B-form DNA with the same sequence. Each RecA monomer interacts with three nucleotides of the DNA (a triplet), and each triplet is also bound by three contiguous RecA monomers. Strikingly, the DNA-RecA interaction, or DNA extension, is not isotropic at the nucleotide level; instead, the DNA comprises a three nucleotide segment with a nearly normal B-form distance between bases (an axial rise of 3.5–4.2 Å for ssDNA and 3.2–4.5 Å for dsDNA), followed by a long, untwisted internucleotide stretch (~7.1–7.8 Å in ssDNA and 8.4 Å in ssDNA) before the next nucleotide triplet, in a repeating pattern. Such an unusual repeat pattern of DNA extension reveals a new structural basis of the dynamics of filament assembly in the presence of ssDNA, including initiation (or nucleation) of filament assembly and the observed cooperativity of RecA-DNA binding.

The RecA-dsDNA-ADP-AlF_4_^-^-Mg^2+ ^crystal structure was postulated to be an end product after the strand exchange reaction between a RecA-ssDNA nucleoprotein filament and a homologous dsDNA target [12,13], implying that RecA protein filaments may complete all functions (including ssDNA binding, donor dsDNA capturing, and strand exchange) in right-handed forms and also within the filament axes. Here, we considered an alternative possibility: that the RecA-dsDNA crystal structures might simply represent annealing products of the ssDNA in the RecA-ssDNA nucleoprotein filament and a complementary ssDNA. First, in the RecA-ssDNA filament structure, the purine and pyrimidine bases of bound ssDNA are outwardly exposed. Second, the complementary ssDNA in the RecA-dsDNA structure makes very few physical contacts with the RecA protein filament, indicating that the annealing of these two ssDNAs has very little impact on the protein structures. Indeed, the overall protein structures of RecA-dsDNA filaments are highly similar to those of RecA-ssDNA-ADP-AlF_4_^-^-Mg^2+ ^structures [12,13]. Because the molecular mechanism of the strand exchange reaction is still not understood, it is important to examine these two different possibilities further.

**Figure 1:**
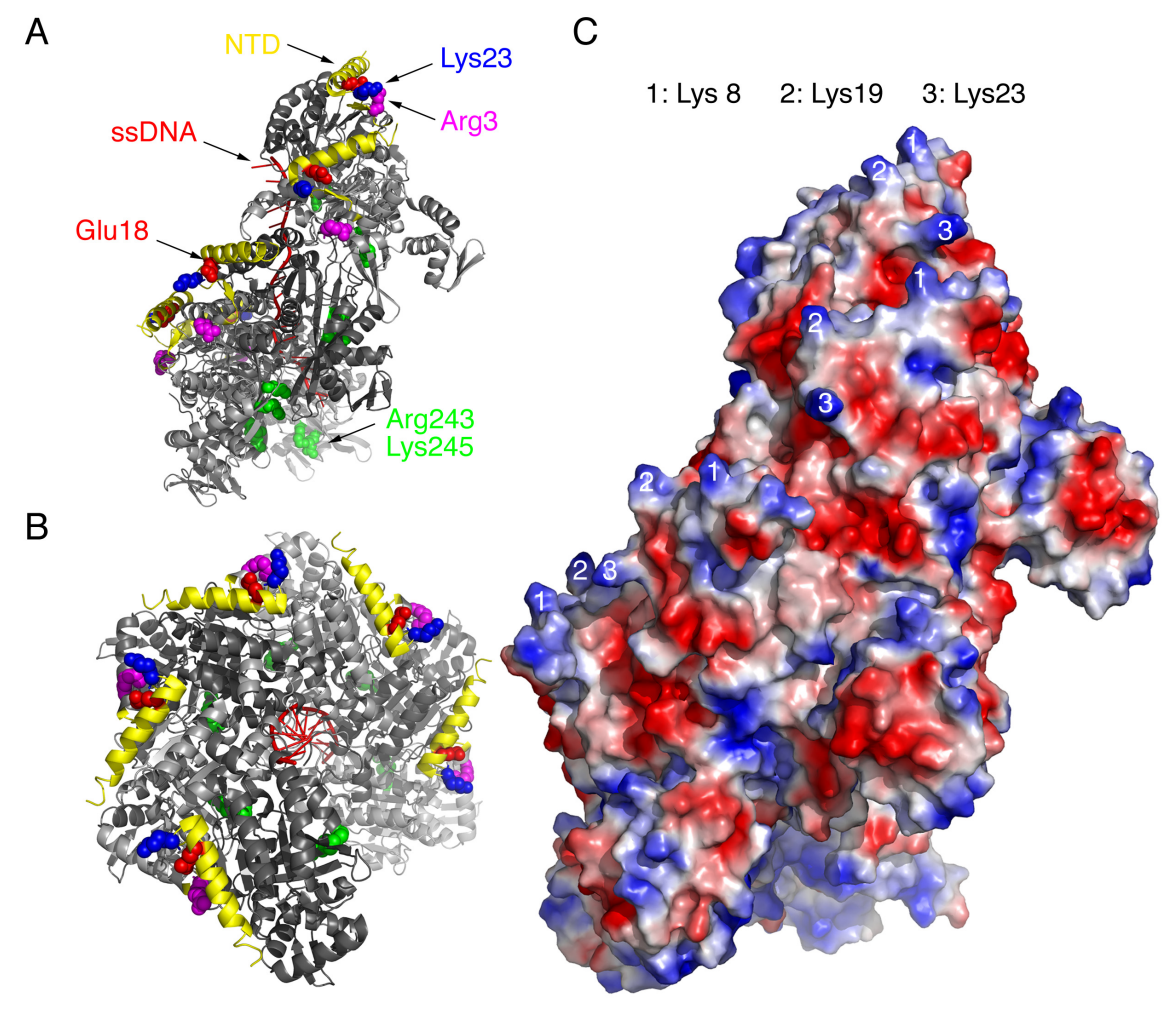
**Structure of a RecA-ssDNA-ADP-AlF_4_^-^-Mg^2+ ^presynaptic nucleoprotein filament**. Shown are ribbon diagrams of side (A) and top (B) views of the recently reported 3CMU crystal structure [12]. The NTDs and ssDNA are shown in yellow and red, respectively. The side chains of three charged residues are depicted as a ball-and-stick model. (C) Surface charge potential of the 3CMU RecA-ssDNA-ADP-AlF_4_^-^-Mg^2+ ^presynaptic nucleoprotein filament. The positively and negatively charged regions are indicated in blue and red, respectively. The positions of Lys8 [1], Lys19 [2], and Lys23 [3] are indicated.

The RecA-ssDNA crystal structures also indicate that Arg243 and Lys245 might constitute a binding site for the donor dsDNA during the strand exchange reaction. Arg243 and Lys245 are ~25 Å away from the filament axis and have a repeat distance of ~28 Å along the filament axis. Their positively charged side chains are solvent-exposed and face towards the central axis of the RecA-ssDNA helical filament (Figure 1A) [12]. This is consistent with previous reports that these positively charged residues are responsible for dsDNA capture during homology pairing and strand exchange [14,15]. However, a closer look at the RecA-ssDNA crystal structure revealed that the positively charged side chains of Arg243 and Lys245 are not exposed to the exterior surface of the nucleoprotein filament; they reside inside the filament (Figure 1B). We speculated that these two amino acid residues might not be solely responsible for dsDNA recruitment. Other structural element(s), located at the outermost surface of the RecA-ssDNA presynaptic filament, may assist the RecA-ssDNA presynaptic filament in its search for donor dsDNA.

In the present study, we report that the NTD of RecA may have such a function. Additional mutant analysis revealed that it might have at least two distinct roles in promoting the RecA-mediated homologous recombination reaction.

## Materials and methods

### RecA protein production, enzymatic assays, and DNA substrates

An improved SUMO fusion protein expression system [16] was used to rapidly produce RecA proteins in *E. coli*. The nuclease assay, D-loop formation assay, ssDNA-dependent ATPase activity assay, and DNA substrates used in this study have also been described in detail [16]. ATPγS (Adenosine 5'-O-(3-thio)triphosphate) and AMP-PNP were purchase from Sigma Aldrich.

### Electron microscopy

The wild-type and mutant *E. coli *RecA proteins (2 μM) were incubated with 4 μM circular ΦX174 dsDNA (in base pairs [bps]) at 37°C for 30 min in reaction buffer (1 mM ATPγS, 10 mM Mg^2+^-acetate, 100 mM Na acetate, 25 mM Tris-Cl pH 7.4), and were chilled on ice to stop the reaction. A droplet (4 μl) was placed on a copper grid (300 mesh, Pelco, USA) coated with fresh carbon for 1 min at room temperature. Excess buffer was then carefully blotted away from the edge of the grid with Whatman #1 filter paper (Whatman Inc., USA). After staining for 4 min with 2.5% uranyl acetate, excess liquid was removed and the samples were air dried at room temperature. Bio-transmission EM was performed with a Tecnai G2 Spirit Bio TWIN (FEI Co., Netherlands) using an acceleration voltage of 120 kV. Images were recorded with a slowscan CCD camera (Gatan MultiScan 600) at a resolution of at least 1024 × 1024 pixels.

### Duplex DNA capture assay

The duplex DNA capture assay was carried out using a protocol modified from that described previously for Rad51 and Hop2-Mnd1 proteins [17]. To obtain presynaptic filaments for the dsDNA capture assay, a 5'-biotinylated ssDNA PA1656 (15 μM in nucleotides) and RecA proteins (5 or 20 μM) were mixed with 4 μL streptavidin-coated magnetic beads (Novagen, USA) in 20 μL of buffer C (20 mM HEPES-KOH at pH 7.0, 1 mM DTT, 100 ng/mL BSA, 2 mM Mg^2+^-acetate, 5% glycerol and 1 mM ATPγS) for 5 min at 37°C. BSA was used as a negative control for RecA-DNA binding reactions. The magnetic beads were isolated with a magnetic separator (Novagen). The supernatant (denoted as "S1") was set aside for later analysis. The magnetic beads were washed twice with 20 mL buffer C, and then mixed with 15 μM of donor dsDNA (bps) in 20 μL of buffer D (20 mM HEPES-KOH at pH 7.0, 1 mM DTT, 11 mM Mg^2+^-acetate, 5% glycerol) at 37°C for 10 min with gentle mixing every 1 min. This donor dsDNA was 300 bps in length, and its central region was homologous to PA1656. The magnetic beads were isolated again with a magnetic separator. The supernatant (denoted as "S2") was set aside for later analysis. After the magnetic beads were washed twice with 20 μL of buffer E (20 mM HEPES-KOH at pH 7.0, 1 mM DTT, 11 mM Mg^2+^-acetate, 5% glycerol and 1 mM ATPγS), proteins and DNA substrates were eluted by incubating with 20 mL of 1% SDS. The SDS eluates (denoted as "B") were separated on either a 12% SDS-PAGE stained with Coomassie blue to visualize bovine serum albumin (BSA) and RecA proteins, or a 1.5% agarose gel stained with ethidium bromide to visualize the donor dsDNA.

## Results

### The NTD of RecA is similar in amino acid sequence to a functional motif of RadA/Rad51/Dmc1

The NTD of *E. coli *RecA contains only 33 amino acid residues and is much smaller in size than the RadA/Rad51/Dmc1 NTDs (> 60 amino acids). The crystal structure of the RecA-ssDNA presynaptic filament reveals that NTDs are located at the exterior surface of the helical filament. Moreover, NTDs are not directly involved in RecA-ssDNA interaction or RecA-ATP binding (Figure 1A) [12]. A superimposition of RecA-RecA pairs from the active RecA-ssDNA presynaptic filament onto an inactive RecA protein filament (achieved by aligning the CADs) revealed a large conformational change in the hinge region (residues 31–40) that connects the NTD and CAD. As a result, these two filaments differ by a 32° rotation and an 18.5 Å translation of the CAD [12]. This hinge region is located immediately after the polymerization motif (*i.e*., Ile26 of *E. coli *RecA), which functions as a fulcrum to mediate a large conformational change in response to ssDNA binding [12]. A similar scenario was also reported in a structural transition of the RecA protein filament from a compressed form to a relaxed form [10]. Intriguingly, RadA/Rad51/Dmc1 protein also contains a similar structural motif that we referred to previously as the subunit rotation motif (SRM) [18]. The SRM also uses the polymerization motif as a fulcrum to mediate rotation along the central axis of the archaeal RadA protein polymer. Continuous clockwise axial rotation of archaeal RadA proteins is responsible for the progression of stepwise structural transitions: first from an inactive ring to a right-handed filament with 6 monomers per helical pitch, then to an overextended right-handed filament with 3 monomers per helical pitch, and, finally, to a left-handed filament with 4 monomers per helical pitch[10,11,18]. A key consequence of these structural transitions is the progressive relocation of the NTDs and the L1 ssDNA binding motif. The NTD has been shown to mediate donor dsDNA binding in both human Rad51 [19] and archaeal RadA [18]. In the RadA right-handed helical filament with 6 monomers per helical pitch, L1 resides near the axis filament and the NTD is located at the exterior surface of the filament. In contrast, in the overextended right-handed helical filament with 3 monomers per helical pitch, L1 relocates to the exterior surface of the filament and constitutes an outward-opening palm structure in combination with the NTD. Inside this palm structure, 5 conserved basic amino acid residues (Lys27 and Lys60 of the NTD; and Arg117, Arg223, and Arg229 of the L1 motif) of *Sulfolobus solfataricus *RadA (*Sso*RadA) surround this pocket (~25 Å in diameter), which may be wide enough to simultaneously accommodate an ssDNA and a donor dsDNA [18]. All five positively charged residues are evolutionarily conserved in all archaeal and eukaryotic RecA family proteins. Therefore, the overextended right-handed filament structure of *Sso*RadA was proposed to represent a structural intermediate during the homologous search and pairing process of archaeal and eukaryotic RecA family proteins [18]. Similarly, as described above, the RecA-ssDNA-ADP-AlF_4_^-^-Mg^2+ ^nucleoprotein filament was also overextended [12,13].

**Figure 2:**
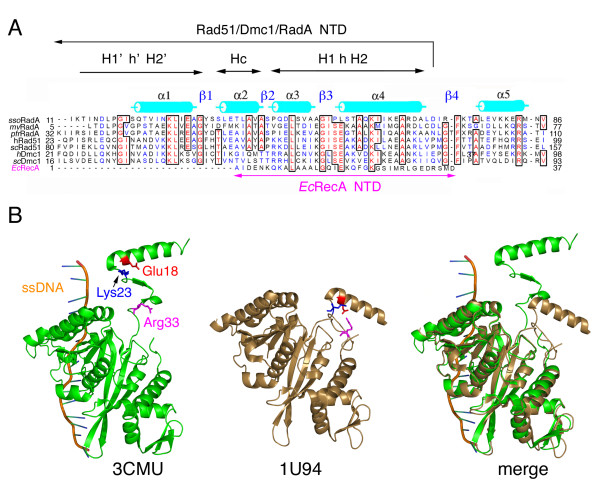
**The E. coli RecA NTD exhibits significant amino acid sequence homology with the NTDs of the homologous proteins Rad51/Dmc1/RadA.** (A) Sequence alignment of the NTDs of RecA proteins from *S. solfataricus *(*Sso*RadA), *M. voltae *(*Mv*RadA), *P. furiosus *(*Pf*Rad51), *H. sapiens *(*Hs*Rad51 and *Hs*Dmc1), *S. cerevisiae *(*Sc*Dmc1 and *Sc*Rad51), and *E. coli *(*Ec*RecA). Secondary structural features of the left-handed *Sso*RadA helical filament are indicated in cyan (α-helices)[10]. Functional motifs are indicated under their corresponding amino acid sequences: the first HhH motif (H'h'H2'), core helix (Hc the), and the second HhH motif (H1Hh2). (B) The NTD carries out a large conformational change in response to ssDNA and ATPγS. Shown are ribbon diagrams of the monomeric RecA structures in the RecA-ssDNA-ADP-AlF_4_^- ^presynaptic filament [12] and in the inactive RecA protein helical filament [3]. The ssDNA and RecA polypeptides are shown in gold and green, respectively. The side chains of Glu18, Lys23, and Arg33 are depicted as ball-and-stick models.

The NTD of RecA was previously considered to be structurally and functionally distinct from those of Rad51/Dmc1/RadA proteins. The RecA NTD contains only a helix (residues 1–23) and a β-loop (residues 24–33). By contrast, the NTDs of archaeal and eukaryotic RecA family proteins are composed of two helix-hairpin-helix (HhH) motifs, in which a pseudo two-fold unit is composed of two HhH motifs linked by a connector α-helix. The HhH motifs and the connector α-helix are denoted as H1'h'H2', H1hH2, and Hc. Each HhH motif contains two helices (denoted as H1, H1', H2, or H2') and a hairpin (denoted as h or h') [18]. We reported earlier that the second H1hH2 motif of *Sso*RadA (*i.e*., the α3-β3-α4 region indicated in Figure 2A) is located at the outer surface of the NTD and constitutes a positively charged patch that is responsible for dsDNA binding. Intriguingly, we found that the α-helix of the RecA NTD (residues 1–23) shows significant amino acid sequence conservation with the second H1hH2 motif of archaeal and eukaryotic RecA family proteins. First, Gly15 and Lys23 of RecA are conserved in all RecA family members listed in Figure 2A. The equivalent respective residues in *Sso*RadA are Gly52 and Lys60, and in human Rad51 are Gly65 and Lys73. Importantly, Gly65 of human Rad51 and Lys60 of *Sso*RadA were both implicated previously in dsDNA binding [18,19]. Second, Lys8 of *E. coli *RecA also conserved in eukaryotic Rad51 and Dmc1 proteins. The positively charged side chains of Lys8, Lys19, and Lys23 all point outward to the outermost surface of the active RecA-ssDNA presynaptic nucleoprotein filament (Figure 1C) [12]. Intriguingly, these three lysine residues are conserved among most, if not all, prokaryotic RecA proteins [20]. Third, Glu18 of RecA is also conserved in several other RecA family proteins (Figure 2A). Glu18 may have a role in RecA interaction with ATP or ssDNA. It forms a salt bridge with Arg33 in a compressed/inactive form of the RecA protein filament [3], (*i.e*., the 1U94 structure, shown in the middle panel of Figure 2B). This salt bridge falls apart in the active/extended RecA-ssDNA-ADP-AlF_4_^-^-Mg^2+ ^presynaptic filaments (3CMU; Figure 2B, right panel) [12]. In the latter case, Glu18 makes hydrogen bonds with Ser25, and Arg33 forms a salt bridge with Glu35. The electrostatic charges of Glu18, Arg33, and Glu36 are diminished or neutralized. As a result, only the positive side chains of Lys8, Lys19, and Lys23 are exposed on the outer surface of the RecA NTD (Figure 1C).

### Production of the wild type and mutant RecA proteins

**Figure 3:**
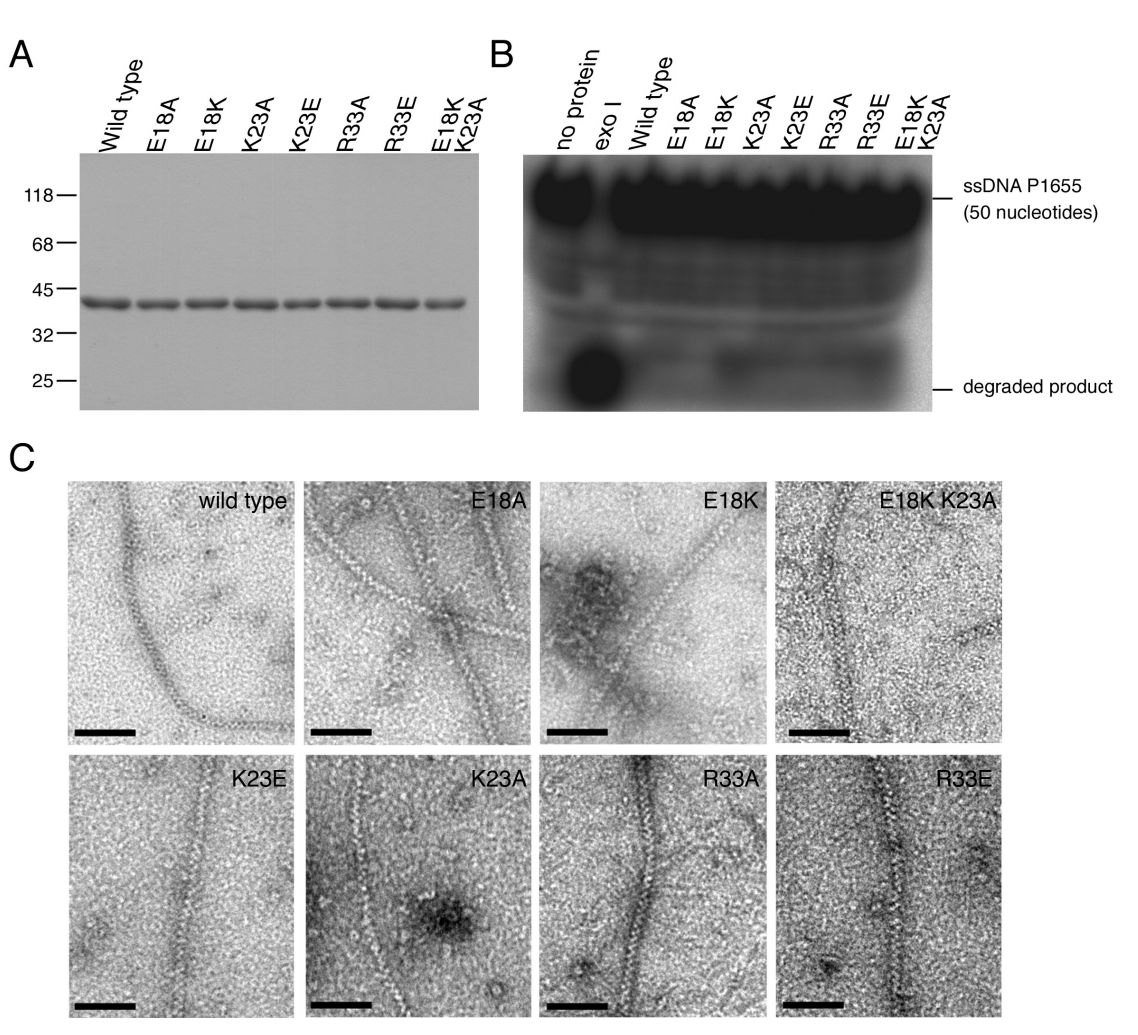
**Production of wild-type and mutant RecA proteins**. An improved SUMO fusion protein expression system was used to produce authentic RecA protein. (A) Purified RecA protein on a 12% SDS-PAGE gel stained with Coomassie Blue. (B) Nuclease activity assay. Purified RecA protein (1 μM) or exonuclease I (20 units; New England Biolabs) was incubated with 5'-end ^32^P-labeled P1656 ssDNA (50 nucleotides, 3 μM), respectively. The reaction mixtures were treated with proteinase K and then electrophoresed on 20% native acrylamide gels. Gels were visualized on a phosphorimager, with overexposure to confirm that the purified RecA proteins exhibited no detectable nuclease activity. (C) Purified RecA proteins are capable of polymerization into nucleoprotein helical filaments. Negative-staining electron microscopy images show various RecA proteins with a ΦX174 dsDNA substrate in the presence of ATPγS. Scale bars (in black) are 100 nm.

To functionally characterize the NTD of RecA, an improved SUMO fusion protein expression system that we developed recently [16] was applied to produce a panel of RecA mutant proteins. Each mutant protein carries one or two point mutations in Glu18, Lys23, or Arg33. To our knowledge, these NTD mutant proteins have not been properly examined before [21]. Native wild-type RecA protein has 352 amino acids, beginning with an alanine. Edman degradation confirmed that the N-terminus of purified wild-type RecA protein was identical to the expected amino acid sequence [16]. Although the purified RecA protein looked reasonably pure (Figure 3A), we still used a 5'-end ^32^P-labeled ssDNA substrate (PA1656, 50 nucleotides) to determine whether it was contaminated with nuclease. *Exo*I was used as a positive control for the nuclease assay, and all 5'-end ^32^P-labeled ssDNA substrates were degraded after incubation with *Exo*I for 30 min. In contrast, no purified RecA proteins cleaved any 5'-end ^32^P-labeled ssDNA substrate under the same conditions (Figure 3B). Therefore, nuclease contamination was not a problem in our production protocol. We confirmed that the purified wild-type RecA was catalytically active in various biochemical assays, and that its activity was indistinguishable from commercially obtained RecA [16]. We also showed by electron microscopy that all NTD mutant proteins examined in this analysis could form helical filaments or protein rings (Figure 3C), indicating they had no apparent defect in polymerization or in formation of the nucleoprotein filament.

### Functional characterization of NTD mutant proteins

**Figure 4:**
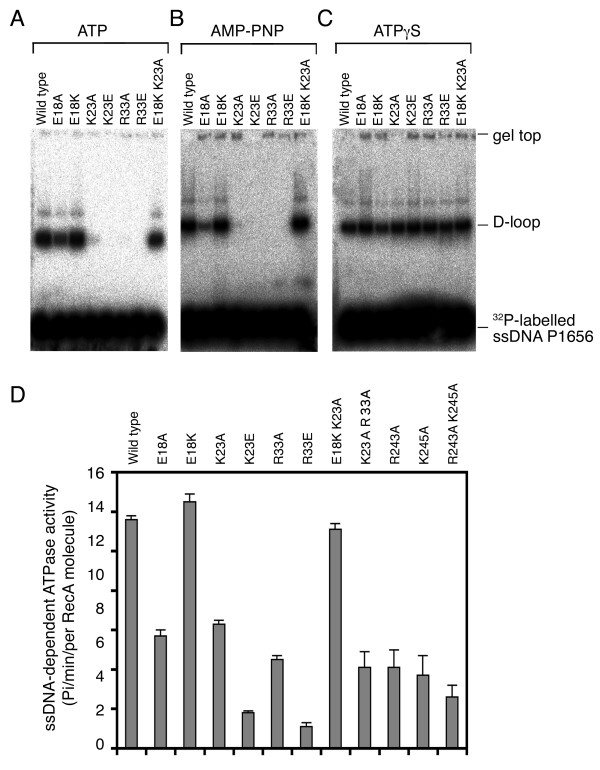
**Functional characterization of wild-type and mutant RecA proteins**. (A-C) D-loop formation. The ability to form a D-loop by RecA proteins was determined in the presence of ATP (A), AMP-PNP (B) or ATPγS (C). Reaction samples from the 10 min time point are shown. (D) The ATPase activities of RecA proteins in response to ssDNA. Wild-type or mutant RecA proteins (0.5 mM) were incubated in the presence of 1 mM Mg^2+^, with or without ΦX174 ssDNA (1 mM nucleotides). ATP hydrolysis was initiated by adding 1 mM ATP (with 0.6 nM [γ-^32^P]ATP) at 37°C. At different time points, 0.3 μL aliquots were withdrawn and spotted on thin layer chromatography paper to separate [γ-^32^P]ATP from ^32^P-labeled inorganic phosphate. All RecA proteins examined here exhibited relatively low ATPase activities (< 0.5 Pi/min/RecA protein) in the absence of ssDNA. Differences between RecA ATPase activities in the presence and absence of ssDNA are shown.

D-loop formation assays were performed with a 5'-end ^32^P-labeled ssDNA PA1656 and a supercoiled dsDNA GW1 as previously described [16]. When ATP or AMP-PNP (a non-hydrolyzable ATP analogue) was used as a nucleotide cofactor, four mutants (K23A, K23E, R33A, and R33E) produced no or very little D-loop product. By contrast, E18A and E18K single mutants, and the E18K/K23A double mutant produced, respectively, ~20%, ~187%, and ~90% of the amount of D-loop products made by wild-type RecA (Figure 4). The gain-of-function phenotype of E18K was strong enough to rescue the K24A mutation under the same conditions. These results suggest that positively charged side chains at the outer surface of the NTD are essential for RecA's function.

**Figure 5:**
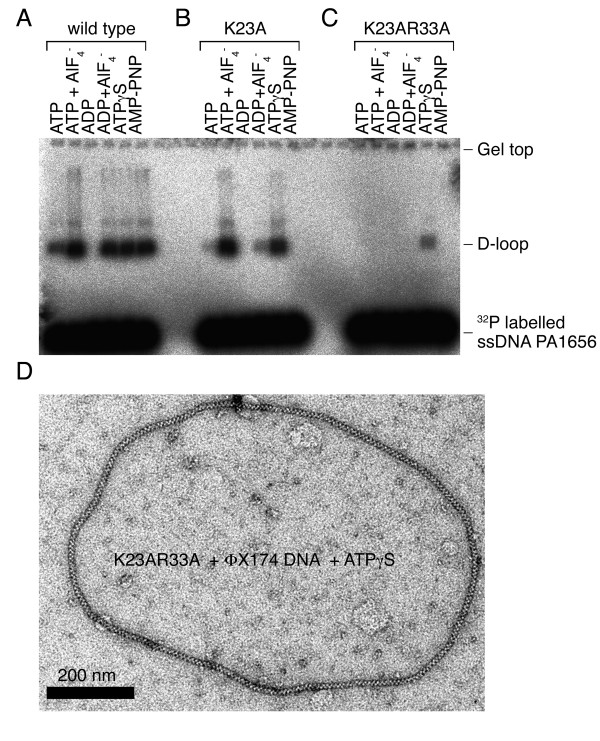
**Effects of different nucleotide cofactors on wild-type (A), K23A (B), and K23A/R33A (C) RecA protein mutants**. D-loop formation in the presence of different nucleotide cofactors, as indicated, is shown. (D) K23A/R33A mutant proteins formed a presynaptic nucleoprotein filament with ssDNA in the presence of ATPγS. Negative-staining electron microscopy image shows K23A/R33A proteins with a circular ΦX174 dsDNA in the presence of ATPγS. Scale bar (in black) is 200 nm.

Surprisingly, when the same assay was performed in the presence of ATPγS, all the RecA-NTD mutant proteins that were examined became catalytically active (Figure 4C). Although ATPγS is a slowly hydrolyzed analog of ATP, ATP hydrolysis alone could not account for the differences between ATP and ATPγS in these RecA-NTD mutants. We also found that K23A (Figure 5) and three other NTD mutants (K23E, R33A, and R33E; data not shown) became catalytically active in D-loop formation upon mixing AlF_4_^- ^with ATP. AlF_4_^- ^was used here to trap the ADP-Pi transition state, because it is able to substitute for inorganic phosphate (Pi) after the hydrolysis of ATP. These results indicate that ATPγS has a higher tendency than AMP-PNP to stabilize the activated transition state of RecA protein. ATPγS and AMP-PNP are known to have different structures among themselves, and in comparison with ATP or ADP-AlF_4_^-^. Interestingly, GTPγS and GMP-PNP also exhibit similar diverse effects to some GTPases. For example, unlike GTPγS, GMP-PNP does not stabilize the activated transition state of Sar1 GTPase [22,23]. Sar1 is a structural component of coat complex II (COPII) vesicle coat, which coordinates the budding of transport vesicles from the endoplasmic reticulum in the initial step of the secretory pathway.

We then speculated that the wild-type, E18K, and E18K/K23A proteins might be better than other NTD mutant proteins at converting ATP into ADP-Pi. Accordingly, we compared the ATPase activities of these three RecA proteins in response to ssDNA. The ssDNA-dependent ATPase activity was determined as described before, by quantification of the inorganic phosphate (Pi) released from γ^32^P-labeled ATP [16]. Indeed, wild-type, E18K, and E18K/K23A proteins all exhibited higher ssDNA-dependent ATPase activity than the other four NTD mutants (K23A, K23E, R33A, and R33E) (Figure 4D).

Taken together, the positively charged residues (Lys23, Arg33, or Lys18 created by the E18K mutation) at the outer surface of the NTD could facilitate the RecA conversion of ATP into intermediate ADP-Pi in response to ssDNA. This conversion could be avoided with the use of ATPγS, ATP-AlF_4_^-^, or even ADP-AlF_4_^-^. ATPγS is known to be better than ATP or AMP-PNP at stabilizing the RecA-ssDNA presynaptic nucleoprotein filament [1,12,24]. However, Lys23 and Arg33 apparently make no direct contact with ssDNA or ATP in the crystal structure of the RecA-ssDNA-ADP-AlF_4_^-^-Mg^2+ ^presynaptic nucleoprotein filament (Figure 1A) [12]. It is likely that these positively charged residues facilitate a RecA protein filament to search for and capture ssDNA in the presence of ATP, and to then convert ATP into intermediate ADP-Pi. As a result, the protein filament binds ssDNA tightly to form an active presynaptic nucleoprotein filament.

### Lys23 and Arg33 are indispensable for RecA's function in the presence of ATPγS

**Figure 6:**
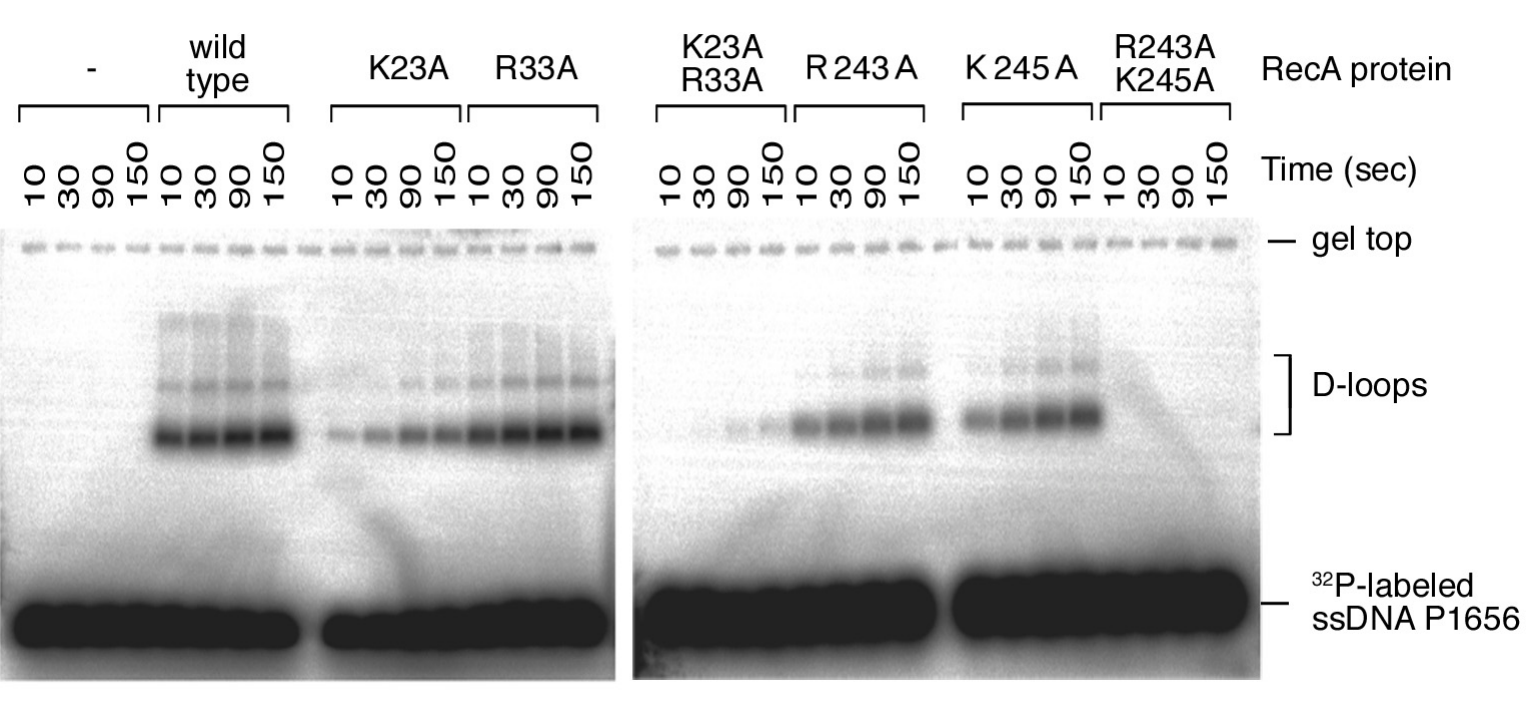
**The K23A/R33A and R243A/K245A double mutants are catalytically inactive in promoting D-loop product formation**. Time course analysis was performed in the presence of ATPγS. Aliquots were withdrawn from the reactions after 10, 30, 90, or 150 seconds, and then processed for analysis.

We generated a K23A/R33A double mutant that, to our surprise, produced far less D-loop product in the presence of ATPγS or ATP-AlF_4_^- ^(Figure 5). However, these three mutants exhibited similar ssDNA binding (data not shown) and ssDNA-dependent ATPase activities (Figure 4D). EM imaging analysis confirmed that this double mutant could still form a nucleoprotein filament with a circular ΦX174 dsDNA, indicating that the K23A/R33A mutant was not defective in RecA polymerization or in formation of nucleoprotein filament (Figure 5D). Therefore, Lys23 and Arg33 together have an additional function in promoting D-loop formation, and this novel function may be similar to those of Arg243 and Lys245. As described above, Arg243 and Lys245 were implicated earlier as binding sites of donor dsDNA [12,14,15,25,26]. We then expressed and purified three additional mutants, R243A, K245A, and R243A/K245A. Like the single mutants, the K23A/R33A and R243A/K245A double mutants exhibited low ATPase activities in response to ssDNA (Figure 4D). We found that the R243A/K245A double mutant, like K23A/R33A, produced very little or no D-loop product in the presence of ATPγS. By contrast, K243A and R245A single mutants, like K23A and R33A, still could produce D-loop products (Figure 6). The latter finding is consistent with a previous report that R243Q and K245N single mutants had only 33% and 66% of the activity, respectively, of the wild-type RecA [15].

### Lys23 and Arg33 are required for the RecA-ssDNA presynaptic nucleoprotein filament to capture dsDNA

**Figure 7:**
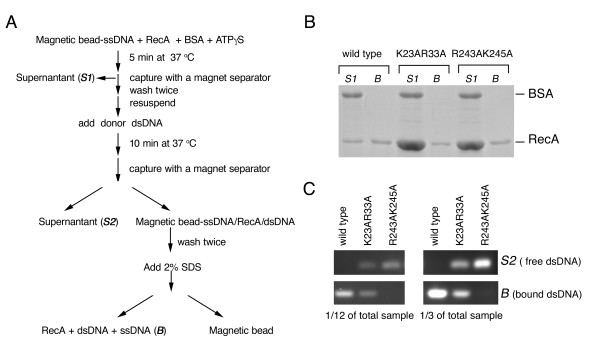
**The presynaptic nucleoprotein filament of K23A/R33A mutant protein is defective in dsDNA capture**. (A) Scheme of assay for examining DNA capture by presynaptic nucleotide protein filaments. See the main text and Materials and Methods for a detailed description. BSA was used as a negative control for ssDNA binding. R243A/K245A was a negative control for dsDNA capture by a presynaptic nucleoprotein filament. (B) SDS-PAGE analysis. RecA and BSA protein in "*S1*" and "*B*" were separated by electrophoresis in a 10% reducing polyacrylamide gel, and visualized by staining with Coomassie blue. (C) DNA agarose gel. For better quantitation, one-third or one-twelfth of the dsDNA (300 bps in length) in "*S2*" or "*B*" were separated on a 1% agarose gel, stained with ethidium bromide and then visualized by UV illumination.

In the D-loop formation reaction, a RecA-ssDNA presynaptic filament must first engage the donor dsDNA molecule for the homology search and heteroduplex formation to occur. We thus performed a dsDNA capture assay according to a protocol (see Figure 7A for schematic) that was modified from one described previously [17]. The R243A/K245A double mutant was used as a negative control in this dsDNA capture assay. RecA, ATPγS, and bovine serum albumin (BSA) were first incubated with magnetic beads coated with 5'-biotinylated PA1656 ssDNA molecules to assemble stable presynaptic filaments. BSA was a negative control for ssDNA binding. To obtain equal amounts of RecA-ssDNA nucleoprotein filaments on the magnetic beads for the subsequent dsDNA capture assay, different amounts of wild-type protein (5 μM) or each double mutant protein (20 μM) were included. The ssDNA-RecA-ATPγS complexes assembled on the magnetic beads were then isolated with a magnetic separator, with the supernatant (denoted as S1) retained for later analysis. The magnetic beads were then resuspended in a buffer containing ATPγS, BSA and a donor dsDNA for 10 min to initiate the homologous pairing or search reaction. This donor dsDNA was 300 bp in length and contained a nucleotide sequence homologous to PA1656 ssDNA. Subsequently, a magnetic separator was used again to separate supernatant (denoted as "S2") and magnetic bead-ssDNA-RecA-dsDNA supercomplexes (denoted as "B"). B was then treated with 1% SDS to elute proteins and nucleic acids, which were then electrophoresed in a 1.5% non-denaturing agarose gel followed by staining with ethidium bromide and UV illumination to quantify the amount of dsDNA captured by the RecA-ssDNA presynaptic filaments. S1, S2 and B were also electrophoresed in a 10% denaturing polyacrylamide gel, followed by staining with Coomassie blue, to quantify RecA and BSA. Notably, S2 contained no or very little RecA protein, because the RecA-ssDNA nucleoprotein filament was very stable in the presence of ATPγS (data not shown). BSA was only detected in the supernatant (S1) and did not bind to magnetic bead-ssDNA (Figure 7B). The DNA-agarose gel results indicate that the wild type RecA-ssDNA nucleoprotein filament was capable of capturing almost all donor dsDNA in the presence of ATPgS (Figure 7C). By contrast, R243A/K245A and K23A/R33A double mutant proteins captured 0% and ~25% of total donor dsDNA, respectively.

Taken together, we conclude that both K23A/R33A and R243A/K245A mutants have two identical defects. First, both mutants had a lower binding affinity for ssDNA than the wild-type protein, because more mutant (20 μM) than wild-type protein (5 μM) was needed to assemble an equal amount of RecA-ssDNA presynaptic nucleoprotein filaments (Figure 7B). Second, once the RecA-ssDNA presynaptic filaments were assembled in the presence of ATPγS, both mutants were defective in capturing donor dsDNA (Figure 7C).

## Discussion

We report here that the *E. coli *RecA NTD exhibits limited but significant amino acid sequence homology with the NTD (specifically, the second H1hH2 motif) of the homologous proteins Rad51/Dmc1/RadA (Figure 2A). Notably, four basic residues (Lys8, Lys19, Lys23, and Arg33) of NTD constitute a positively charged helical patch along the outer surface of the RecA-ssDNA-ADP-AlF_4_^-^-Mg^2+ ^nucleoprotein filament. We also found that these four basic residues at the NTD have at least two distinct roles in promoting RecA-mediated homologous recombination by capturing DNA. First, they help the RecA protein, in response to ssDNA, to convert ATP into intermediate ADP-Pi, a process that can be avoided by use of ATPγS or ATP-AlF_4_^-^. Then, the NTD facilitates the RecA-ssDNA presynaptic nucleoprotein filament capture of donor dsDNA during the homologous search reaction. We think this function may be similar to those of the NTDs in eukaryotic and archaeal RecA proteins [18,19].

The second function of the *E. coli *RecA NTD may be similar to that of Arg243 and Lys245. The NTD and Arg243/Lys245 might function at the same time to facilitate an active RecA-ssDNA-ATPγS nucleoprotein filament to capture dsDNA. However, judging from the crystal structure of the RecA-ssDNA-ADP-AlF_4_^-^-Mg^2+ ^nucleoprotein filament (Figure 1) [12], it is unlikely for a donor dsDNA to simultaneously contact both Lys23/Arg33 and Arg243/Lys245. This is because the basic amino acid residues of the NTD (including Lys8, Lys19, Lys23, and Arg33) are located along the outermost surface of the nucleoprotein filament, while Lys243 and Arg245 are embedded in the center of the nucleoprotein filament (Figure 1B). Therefore, we propose the following model to explain their functions during D-loop formation reaction. After an active RecA-ssDNA nucleoprotein filament is assembled in the presence of ATPγS (or ADP-Pi), the positively charged amino acid residues of the NTD will first non-specifically contact the phosphate groups of dsDNA, subsequently helping Lys243 and Arg245 to establish interactions with this donor dsDNA, and then carry out the strand exchange reaction. A prerequisite for this hypothesis is a rather large structural movement between the NTD and the CAD. This is not impossible. In fact, two similar incidents have been reported recently. First, during assembly of the RecA-ssDNA nucleoprotein filament, a hinge region that connects the NTD and CAD can undergo a large conformational change in response to ssDNA and ADP-AlF_4_^-^-Mg^2+^. This hinge region is located immediately after the polymerization motif, which functions as a fulcrum to mediate this large conformational change [12]. Second, we reported that clockwise axial rotation of the SRM of archaeal RadA proteins is responsible for a serial structural transition of RadA polymers from a protein ring to a right-handed helical filament, then to an over-wound right-handed helical filament, and finally to a left-handed helical filament. The SRM is located between the NTDs and CADs of RadA/Rad51/Dmc1 proteins, and it also uses the polymerization motif as a fulcrum to mediate this clockwise rotation along the helical filament axis [10,11,18]. It would be interesting to investigate whether the RecA NTD actually completes the rest of the dsDNA capture by rotating along the central axis of a presynaptic nucleoprotein filament.

Our results here are consistent with several previous studies on the RecA NTD. First, modeling of a 24-residue RecA N-terminal peptide revealed that the four positively charged residues (Lys6, Lys8, Lys19, and Lys23) were capable of binding DNA phosphate groups by electrostatic interactions [27]. Second, it was reported that K6A/K19A and K6A/K23A double mutants both exhibited severe defects in RecA function *in vivo *[28]. Third, deletion of the first N-terminal 9 amino acids of RecA (RecA-Δ9) manifested a rec^- ^phenotype [29]. The RecA-Δ9 protein loses both Lys6 and Lys8, although the authors emphasized only the lack of Lys6. They then postulated that the Δ9 mutant was defective in mediating essential monomer-monomer contact [29], because the ε-NH_3 _group of Lys6 forms a salt bridge with the carboxylate oxygen of the Asp139 side chain in the neighboring subunit [3]. This hypothesis was correct for the free RecA protein filament, because the purified K6A mutant protein was indeed partly defective in oligomeric interaction in the absence of DNA [30]. The Lys6-Asp139 salt bridge also exists in the RecA-ssDNA-ADP-AlF_4_^-^-Mg^2+ ^nucleoprotein filament [12]. However, the K6A mutant protein demonstrated apparently normal formation of RecA-ssDNA helical filaments *in vitro *[30]. In addition, the *recA*-K6A and *rec*A-K6D mutants both exhibited a rec^+ ^phenotype [30]. Therefore, a decrease in monomer-monomer contact due to breakage of the Lys6-Asp139 salt bridge is not likely to be the primary cause for the rec^- ^phenotype of Δ9, K6A/K19A, and K6A/K23A mutants. Since the basic side chains of Lys8, Lys19, and Lys23 are located at the outer surface of RecA or RecA-ssDNA filaments to bind ssDNA or dsDNA, we think the rec^- ^phenotype of Δ9, K6A/K19A, and K6A/K23A mutants may be the result of a loss or mutation of Lys8, Lys19, or Lys23, respectively.

In conclusion, we showed in this report that the NTD of RecA protein sequentially mediates ssDNA and dsDNA binding during homologous recombination. Our results also imply that a rather large rigid body movement between the NTD and CAD may be required for an active RecA-ssDNA-ATPγS-Mg^2+^nucleoprotein filament to carry out homology searching and binding to a target dsDNA. We suggest that such a rigid body movement is mediated by axial rotation of the NTD and CAD along the central axis of the RecA protein filament.

## Competing interests

The authors declare that they have no competing interests.

## Authors' contributions

CDL carried out all experiments and analyzed the data. TFW conceived and designed the experiment, analyzed the data, wrote the paper and the principle investigator. Both authors read and approved the final manuscript.
